# Prophylactic supplementation of resveratrol is more effective than its therapeutic use against doxorubicin induced cardiotoxicity

**DOI:** 10.1371/journal.pone.0181535

**Published:** 2017-07-20

**Authors:** Heba Samy Shoukry, Hania Ibrahim Ammar, Laila Ahmed Rashed, Maha Balegh Zikri, Ashraf Ali Shamaa, Sahar Gamal Abou elfadl, Ejlal Abu-Al Rub, Sekaran Saravanan, Sanjiv Dhingra

**Affiliations:** 1 Department of Physiology, Biochemistry and Histology, Faculty of Medicine, Cairo University, Cairo, Egypt; 2 Department of Surgery, Faculty of Veterinary Medicine, Cairo University, Giza, Egypt; 3 St. Boniface Hospital Research Centre, Department of Physiology, University of Manitoba, Winnipeg, Canada; University of Central Florida, UNITED STATES

## Abstract

Resveratrol (RSV), a polyphenolic compound and naturally occurring phytoalexin, has been reported to exert cardio-protective effects in several animal studies. However, the outcome of initial clinical trials with RSV was less effective compared to pre-clinical studies. Therefore, RSV treatment protocols need to be optimized. In this study we evaluated prophylactic versus therapeutic effect of resveratrol (RSV) in mitigating doxorubicin (Dox)-induced cardiac toxicity in rats. To investigate prophylactic effects, RSV was supplemented for 2 weeks along with Dox administration. After 2 weeks, Dox treatment was stopped and RSV was continued for another 4 weeks. To study therapeutic effects, RSV treatment was initiated after 2 weeks of Dox administration and continued for 4 weeks. Both prophylactic and therapeutic use of RSV mitigated Dox induced deterioration of cardiac function as assessed by echocardiography. Also RSV treatment (prophylactic and therapeutic) prevented Dox induced myocardial damage as measured by cardiac enzymes (LDH and CK-MB) in serum. Which was associated with decrease in Dox induced myocardial apoptosis and fibrosis. Interestingly our study also reveals that prophylactic use of RSV was more effective than its therapeutic use in mitigating Dox induced apoptosis and fibrosis in the myocardium. Therefore, prophylactic use of resveratrol may be projected as a possible future adjuvant therapy to minimize cardiotoxic side effects of doxorubicin in cancer patients.

## Introduction

Doxorubicin (Dox) is an effective broad spectrum anti-neoplastic agent. The therapeutic index of this highly potent anti-cancer drug is reduced due to cardio-toxic side effects [1,2]. Dox induced deterioration of cardiac function mainly occurs due to myocardial apoptosis [3,4]. Doxorubicin induces cardiac cell apoptosis via intrinsic and extrinsic pathways [5,6]. Both these pathways are linked and that molecules in these two pathways influence each other. Caspase 3 is one such molecule which is involved in both intrinsic and extrinsic pathways [7,8]. Caspase 3 protein is a member of cysteine aspartic acid protease family. The nuclear factors of activated T-cells (NFATs) family members are transcriptional factors that play an important role in cardiac and skeletal functions [9]. The deletion of NFAT1 gene during embryogenesis causes lethal cardiac defects [10,11]. On the contrary, activation of NFAT 2,3 in a Ca/calcenurin-dependent manner mediate adverse remodeling and apoptosis [12–14].

Resveratrol (RSV: trans-3,5,4′-trihydroxystilbene), a polyphenolic compound and naturally occurring phytoalexin was shown to exert strong cardioprotective actions. RSV prevented H2O2 induced oxidative stress in adult cultured cardiomyocytes [15]. Also RSV administration exerted cardioprotective effects by reducing oxidative stress in a rat model of diabetic cardiomyopathy [16]. Similarly, RSV inhibited hypoxia-induced apoptosis in myocardial cells by preventing an increase in Bax and caspase 3 activation [17–20]. Resveratrol also prevents Dox-induced cardiotoxicity by mitigating cardiomyocyte apoptosis, oxidative stress and cardiac fibrosis [21–23]. The cardio protective effects of RSV are reported to be mediated by different cell signaling pathways, including AMP-activated Protein Kinase and Akt survival pathways [24,25]. The beneficial effects of RSV administration in experimental models of cardiac complications have been reported in several animal models [26–28]. However, the outcome of initial clinical trials in cardiac patients suggested marginal benefits after RSV supplementation [29,30]. Therefore, treatment protocols and timing of RSV supplementation during cardiac injury need to be optimized. In this study we aimed to compare potential preventive and therapeutic role of RSV in an experimental model of Dox induced cardiotoxicity. In the current study we also sought to investigate the mechanisms of RSV mediated improvement in cardiac function.

## Materials and methods

### Animals

Adult male Wister rats (140-150g), (n = 32) were used for the current study, all the animals were kept in the animal care facility of the Cairo University and were provided ordinary rat chow and water ad libitum with a 12 hrs light-dark cycle. The experimental protocol and procedures were approved by the Institutional Animal Care and Use Committee of the Cairo University. Animals were allowed to acclimatize for 10 days prior to the start of study.

### Animal grouping and treatment protocol

Animals were randomly allocated into four groups; Control group (*n* = 8): received vehicle solution, saline (0.2 ml ip); Dox group (n = 8): injected with Dox (ADRICIN, EIMC United Pharmaceuticals, Cairo) in six equal doses (cumulative dose 2.5 mg/kg body wt, ip) over a period of 2 weeks; RSV-Dox group (*n* = 8): in order to determine the prophylactic effect of resveratrol in preventing the Dox induced cytotoxicity, this group received both Dox (2.5 mg/kg body wt, ip) and RSV (Sigma Aldrich, USA, at a dose of 20mg/kg body wt, orally dissolved in saline) for 2 weeks, after 2 weeks Dox was stopped, and RSV was continued for next 4 weeks; Dox–RSV group (*n* = 8), in order to investigate the therapeutic effect of RSV, this group received Dox (2.5 mg/kg body wt, ip) for 2 weeks, then Dox treatment was stopped and RSV treatment (20mg/kg body wt, orally dissolved in saline) was initiated and continued for 4 weeks.

Animals were monitored for body weight at the beginning, 2weeks and 6weeks from the start of experiments. At the end of the study, rats were given halothane inhalation anesthesia, after confirmation of deep anesthesia, hearts were removed surgically and divided for histopathological and biochemical analysis.

### Heart function

Echocardiography was performed in different groups at the beginning, after 2 weeks of Dox administration and at the end of the experimental period (6 weeks). Two –dimensional and M-mode recording of short axis view was performed using a 8-10MHz liner transducer (maximum depth of 3 cm) attached to an ultra-sonographic machine (Samsung Madison, SONOACE-R3-Korea). The following measurements were recorded: left ventricular internal dimension at end-diastole (LVIDd), left ventricular internal dimension at end-systole (LVIDs), fractional shortening (FS%) and ejection fraction (%EF).

### Serum cardiac enzymes

Blood samples were collected at the beginning and at the end of the study (6 weeks) to measure cardiac enzymes creatine kinase-MB (CK-MB) and lactate dehydrogenase (LDH) in serum. Briefly, blood samples were collected in dry test tubes and allowed to coagulate at room temperature for 30 min. Serum was separated by centrifugation at 3000 rpm for 10 min. The cardiac enzymes CK-MB and LDH were measured by commercial kits (Stanbio Laboratory, Boerne, TX, USA).

### NFAT3 and NFAT5 levels

Total RNA was extracted from homogenized rat tissues using the RNAeasy mini kit (Qiagen, Germany). cDNA synthesis was performed using a superscript kit from Invitrogen and RT-PCR was performed using a Step One Plus^™^ sequence detection system from Applied Biosystems (Germany). The sequences of PCR primer pairs used for each gene are presented in Table 1. Relative expression of NFAT3 and NFAT5 were normalized to the housekeeping gene ß-actin.

**Table 1:** List of primers used for RT-PCR analysis.

Gene	Primers sequence (5′-3′)		Gene bank accession number	Product Length
NFAT 3	Forward	CCACCAACTGCTCTGACTGC	NM_001108447.1	365
	Reverse	CCTAGCTATGCAACCAGGTCAC		
NFAT5	Forward	AGTGGATGCCAGAGTGTTGTC	NM_001107425.1	237
	Reverse	CGAACAGAAGCCACCACACA		
ß-actin	Forward	TATCCTGGCCTCACTGTCCA	NM_031144.3	120
	Reverse	AACGCAGCTCAGTAACAGTC		

### Histopathological analysis

Hematoxylin and eosin (H&E) staining was performed to assess myocardial damage (Kiernan 2001). Briefly cardiac tissue samples from all the groups were fixed in 10% formalin for 48 hours and paraffin blocks were prepared. Each sample was cut into 5μm thick sections and taken onto poly-lysine coated slides. Each slide was then deparaffinized in xylene, rehydrated in different descending concentrations of ethanol and stained in hematoxylin. Next, the slides were counter stained in eosin, dehydrated in increasing concentrations of ethanol as well as xylene and mounted. The images were captured under the microscope. Using Leica Qwin 500 LTD computer assisted image analysis software (Cambridge, UK) assessment of the area of degenerated (deeply acidophilic) cardiac myocytes was measured in H&E stained sections. The measurements were done in 10 high power fields (HPF).

To assess myocardial tissue fibrosis, heart sections were stained with Masson’s Trichrome (Bancroft and Gamble 2008) and % area of collagen fibres was calculated using Leica Qwin 500 LTD computer assisted image analysis software (Cambridge, UK).  

### Immunohistochemistry

Serial sections were cut and taken onto poly-lysine coated slides. Tissue sections were boiled in 10 mM citrate buffer pH 6.0 for 10–20 minutes and then left to cool at room temperature for 20 min. After washing twice in phosphate buffer saline (PBS), sections were incubated with 0.1 ml of rabbit anti-caspase 3 (3015–100, Biovision, USA) antibody. After 1hr of incubation with primary antibody slides were washed with PBS and biotin labelled secondary antibody was applied for 10 minutes at room temperature. After washing slides were incubated with DAB chromogen mixture for 10–15 minutes at room temperature. The slides were counterstained with Mayer-Haematoxylin for 1–3 minutes. Tonsils specimens were used as positive controls. Negative controls were myocardial sections without primary antibody. The images were captured under the microscope. Using Leica Qwin 500 LTD computer assisted image analysis software (Cambridge, UK) caspase 3 positive area (area%) was measured in the sections. The measurements were done in 10 high power fields (HPF).

### Bax and Bcl-xl levels

Bax and Bcl-xl protein levels were measured by Western blotting. Briefly, myocardial tissue protein extracts were prepared from control and treated samples in different groups, and suspended in PBS containing protease inhibitor cocktail. 30μg of protein was loaded onto 10% FastCast Acrylamide gel (Bio-Rad Laboratories Ltd). SDS-PAGE electrophoresis, immunoblotting, and protein detection were done for Bax, Bcl-xl and ß-actin using anti Bax (sc-493, Santa Cruz), anti Bcl-xl (sc-8392, Santa Cruz) and anti ß-actin (sc-47778, Santa Cruz) antibodies. Band intensity was analyzed by ChemiDoc^™^ imaging system with Image Lab^™^ software version 5.1 (Bio-Rad Laboratories Inc., USA). The results were normalized with ß-actin.

### Statistical analysis

Data is presented as mean±SD. One-way analysis-of-variance (ANOVA) followed by Bonferroni post-hoc test was performed using SPSS software. p values <0.05 were considered statistically significant.

## Results

### Body weight

A significant increase (*P* < 0.05) in the body weight in control group, RSV-Dox and Dox-RSV groups was observed after 2 and 6 weeks of treatment. However, we found a decrease in the body weight in Dox administered animals after 6 weeks of treatment (Table 2).

**Table 2:** Body weight (in grams) in different groups measured at baseline, after 2 and 6 weeks.

	Body weight (gms)			
	Control	Dox	RSV-Dox	Dox-RSV
Baseline	144.28±4.94	146.66±4.71	144 ±4.89	144± 4.47
2 weeks	166.6±4.67*	153.33±7.90	166.6±4.71*	167.6 ±4.71*
6 weeks	168.57±13.55*	124.2±4.94^#^	170±5.34*	178.5± 6.38*

Data are mean ± SD,

*P < 0.05, significantly different from respective baseline group,

^#^*P* <0.05, compared to baseline in Dox group.

### Echocardiography

**Fig 1:**
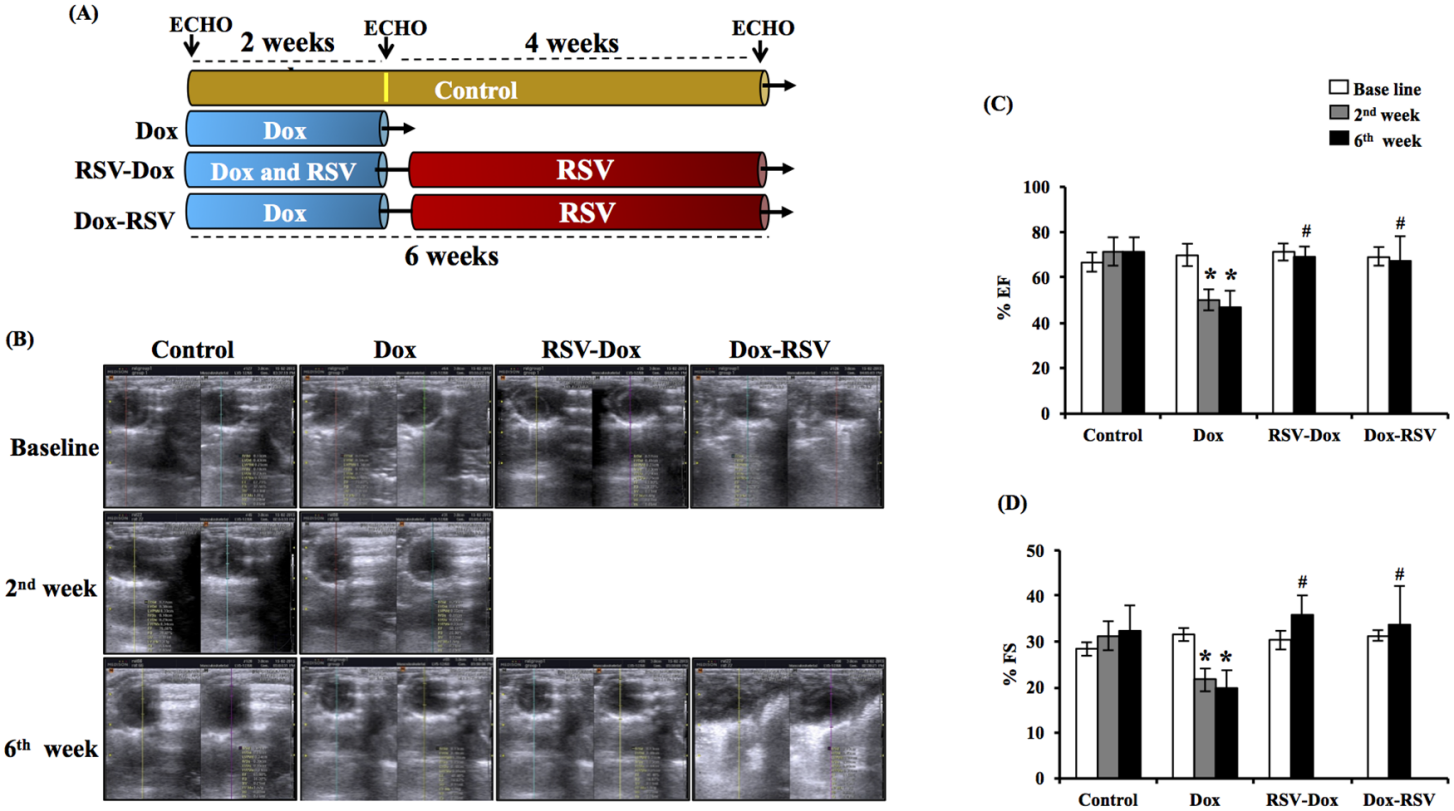
Effect of Dox, RSV-Dox and Dox-RSV on %EF, %FS was measured by echocardiography. RSV treatment prevented Dox induced deterioration of cardiac function. (A) Graphical representation of time points for treatment protocol. (B). Representative M-mode images from different groups. (C). % Ejection fraction (%EF). (D). % Fractional shortening (%FS). Dox treatment for 2 weeks significantly decreased both %EF and %FS, RSV treatment along with Dox or after 2 weeks of Dox supplementation mitigated Dox induced deterioration of cardiac function. Data are mean ± SD, *P < 0.05, significantly different from respective control group, #*P* <0.05, compared to respective Dox group.

Dox induced deterioration of cardiac function was evident after 2 weeks of drug administration with a significant decrease in %EF and %FS (Fig 1). Cardiac function further deteriorated at 6 weeks of Dox administration. Supplementation of RSV along with Dox prevented Dox induced deterioration of heart function. Also RSV treatment for 4 weeks following Dox therapy prevented Dox induced deterioration of cardiac function (Fig 1A–1D).

### NFAT3 and NFAT5

**Fig 2:**
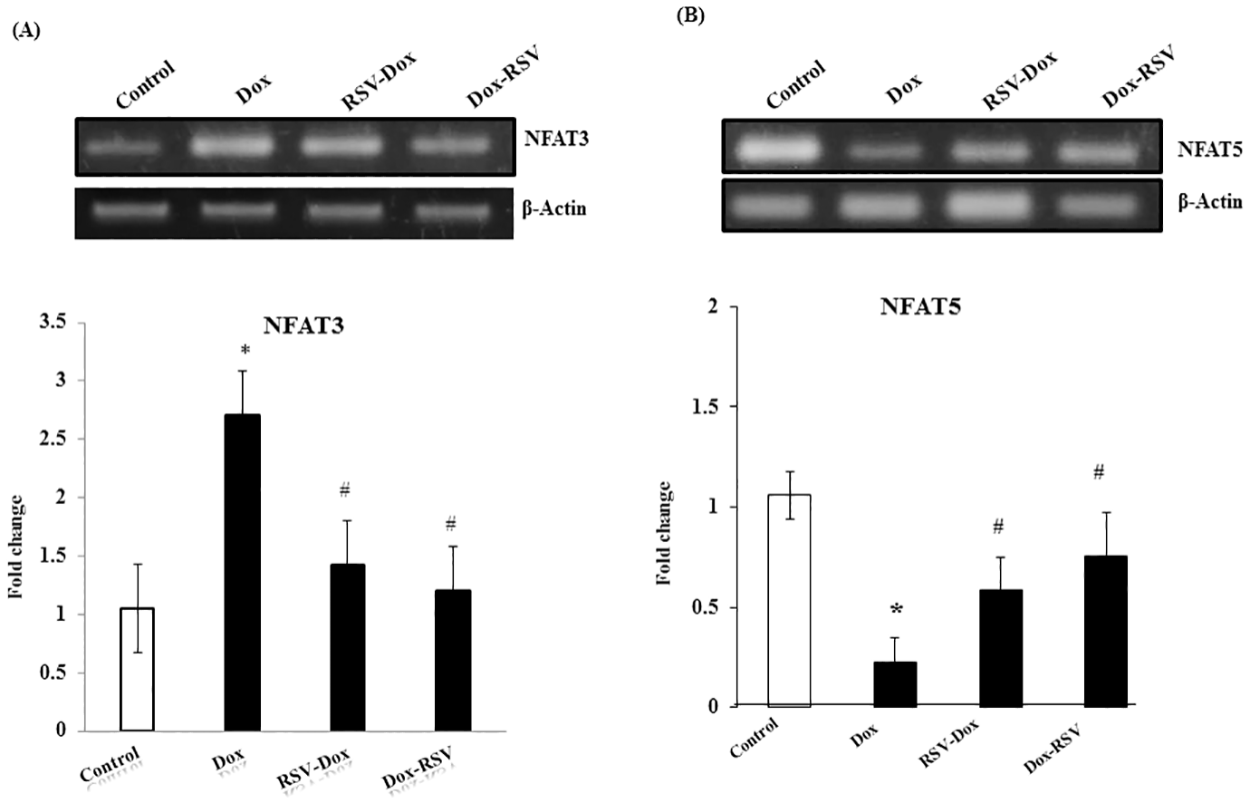
Effect of Dox, RSV-Dox and Dox-RSV on NFAT 3 and NFAT5 expression. (A&B) NFAT3 and NFAT5 expression was measured by RT-PCR. Dox treatment for 2 weeks significantly increased NFAT3 (A) and decreased NFAT5 (B), RSV supplementation along with Dox or after 2 weeks of Dox treatment prevented NFAT3 increase and NFAT5 decrease. ß-actin was used as internal control. Data are mean ± SD. *P < 0.05, significantly different from respective control group, #*P* < 0.05, significantly different from respective Dox group.

NFAT3 and NFAT5 levels were measured by RT-PCR. We found a significant increase in NFAT3 in Dox group that was mitigated by co-treatment with RSV. Resveratrol treatment after 2 weeks of Dox administration also prevented NFAT3 increase. However, NFAT5 levels were significantly decreased in Dox group, co-treatment with RSV or RSV treatment after 2 weeks of Dox administration increased NFAT5 levels (Fig 2A and 2B).

### Histological examination

#### H&E staining

**Fig 3:**
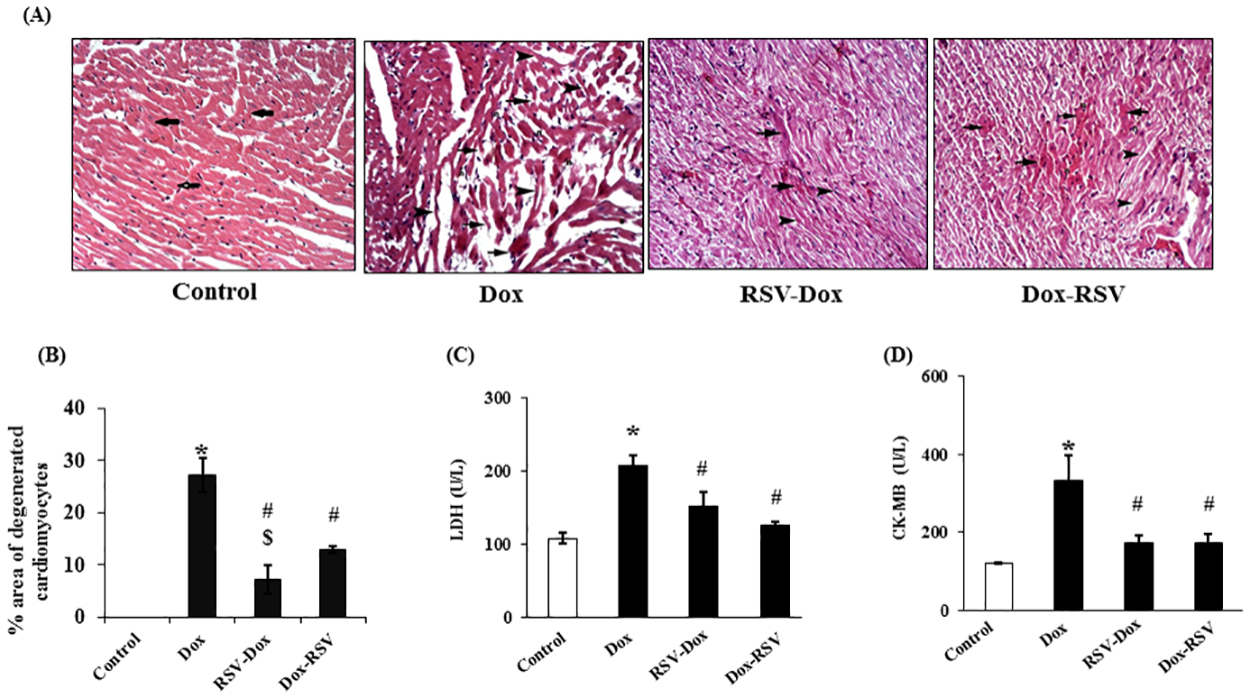
Resveratrol treatment rescued Dox induced myocardial damage. (A). H & E staining: Photomicrographs of rat myocardial sections (x 200). Control group showed muscle fibers arranged in different directions (arrows), Dox group showing a wide area of widely spaced deeply acidophilic fibers including multiple disrupted (arrows), multiple thin attenuated (arrowheads) and multiple fibers exhibiting dark peripheral nuclei. RSV-Dox showed few congested blood vessels among muscle fibers, few deeply acidophilic (arrows) and few thin attenuated fibers (arrowheads) were present. In Dox-RSV group also sections showed some congested blood vessels among apparently normal muscle fibers, in addition to some deeply acidophilic fibers (arrows), some thin attenuated (arrowheads) and some fibers exhibiting dark peripheral nuclei were detected. (B). Quantitative analysis of (percent area) degenerated myocytes. (C&D). LDH and CK-MB levels in serum measured by commercial kits purchased from Stanbio Laboratory USA. Data are mean ± SD. *P < 0.05, significantly different from respective control group, #*P* < 0.05, significantly different from respective Dox group, ^$^*P* < 0.05, significantly different from respective Dox-RSV group.

Control sections revealed normal architecture of cardiac muscle fibers arranged in different directions. Myocardial sections in Dox group showed wide areas of widely spaced deep acidophilic fibers including multiple disrupted and thin attenuated fibers exhibiting dark peripheral nuclei (Fig 3A and 3B). In RSV-Dox group few congested blood vessels were detected among muscle fibers, besides few deeply acidophilic and few thin attenuated fibers were detected. In Dox-RSV group also some congested blood vessels were evident among muscle fibers. In addition, some deeply acidophilic fibers, some thin attenuated and some fibers exhibiting dark peripheral nuclei were seen compared to Dox group (Fig 3A and 3B). Interestingly when we compared the % area of degenerated cardiomyocytes in both RSV-Dox and Dox-RSV groups, resveratrol performed better when it was supplemented prophylactically compared to its therapeutic use.

#### Masson's trichrome staining

**Fig 4:**
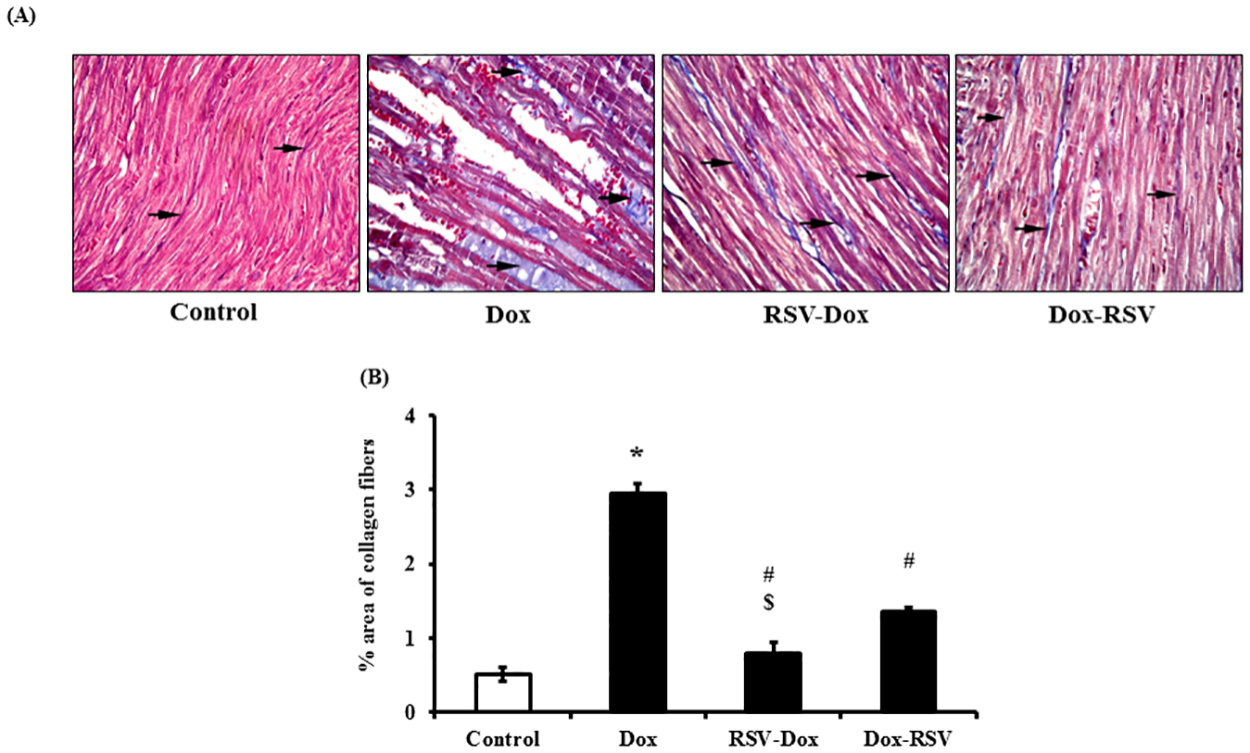
Resveratrol treatment mitigates Dox induced cardiac fibrosis. (A) Masson’s trichrome staining: Photomicrographs of rat myocardial sections (X 200): Control group showed fine collagen fibers (arrows) among muscle fibers. Dox group showed dense collagen fibers (arrows) among thin muscle fibers. RSV-Dox group showed fine collagen fibers (arrows) among muscle fibers. In Dox-RSV group lesser amount of dense collagen fibers (arrows) among muscle fibers were detected. (B) Quantitative analysis (percent area) of collagen fibers. Data are mean ± SD. *P < 0.05, significantly different from respective control group, #*P* < 0.05, significantly different from respective Dox group, ^$^*P* < 0.05, significantly different from respective Dox-RSV group.

In control group fine collagen fibers were found among muscle fibers. In Dox group, dense collagen fibers were observed among thin fibers. In both RSV-Dox and Dox-RSV groups, we observed decreased level of fibrosis, as there was lesser amount of collagen deposition among muscle fibers in RSV treated groups. However, prophylactic use of RSV was more effective than its therapeutic use in mitigating Dox induced fibrosis, as % area of collagen deposition was significantly greater in Dox-RSV group compared to RSV-Dox group (Fig 4A and 4B).

### Cardiac enzymes

Both CK-MB and LDH are markers of cardiac damage after myocardial infarction. In the current study we found a significant elevation in CK-MB and LDH in the blood from Dox treated rats. Treatment with RSV along with Dox therapy or after 2 weeks of Dox administration decreased the levels of both CK-MB and LDH (Fig 3C and 3D).

### Myocardial apoptosis

**Fig 5:**
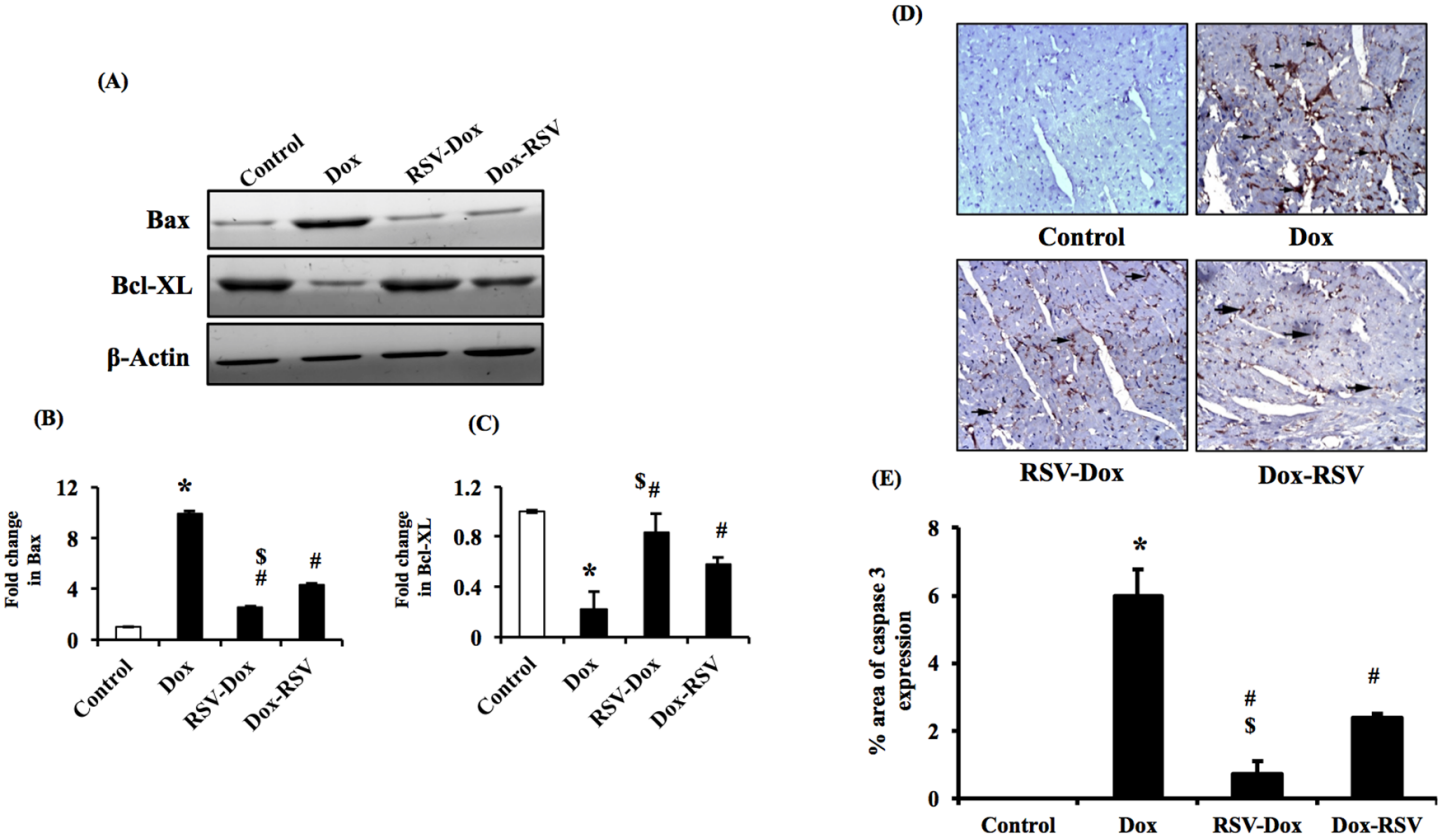
Resveratrol treatment prevents Dox induced myocardial apoptosis. (A, B & C) Bax and Bcl-xl protein levels by Western blot. The protein levels of Bax increased and Bcl-xl decreased in Dox group compared to control animals. Treatment with RSV along with Dox or RSV treatment following Dox administration prevented Dox induced increase in Bax, and decrease in Bcl-xl levels. Histograms depict densitometric analysis (B and C). The results were normalized to ß-actin. (D & E) Caspase 3 expression by immunohistochemistry: Photomicrographs of rat myocardial sections (X 200). Control group showed –ve immunostaining among muscle fibers. Dox group showed caspase3 +ve areas (arrows) in myocardial sections. RSV-Dox group showed decreased expression of caspase 3 in myocardial sections. In Dox-RSV group also there was a decrease in caspase 3 +ve area. Data are mean ± SD. *P < 0.05, significantly different from respective control group, #*P* < 0.05, significantly different from respective Dox group, ^$^*P* < 0.05, significantly different from respective DOX-RSV group.

Myocardial apoptosis was detected by measuring the levels of pro-apoptotic protein Bax and anti-apoptotic protein Bcl-xl. Our data demonstrate that protein levels of Bax increased and Bcl-xl decreased in Dox group compared to control. However, co-treatment with RSV along with Dox or RSV treatment following Dox administration prevented Dox induced increase in Bax and decrease in Bcl-xl levels (Fig 5A–5C). We also performed immunohistochemistry to measure caspase 3 expression in myocardial tissue. Caspase 3 is a well-known marker for cellular apoptosis, in control group, we could not detect any caspase 3 positive areas (Fig 5D and 5E). Whereas, in Dox group, there was a significant increase in caspase 3 expression. Therefore, Dox treatment lead to an increase in myocardial apoptosis. Co-treatment with RSV along with Dox or RSV treatment following Dox administration was associated with decrease in myocardial apoptosis, as there was a downregulation of Dox induced increase in caspase 3 expression in both RSV-Dox and Dox-RSV groups (Fig 5D and 5E). However, prophylactic use of RSV was more effective than its therapeutic use in mitigating Dox induced apoptosis.

## Discussion

Several studies in animal models have reported cardio-protective effects of resveratrol [21, 26–28]. Based on the encouraging outcome of these preclinical studies, various clinical trials were conducted recently [29–31]. The outcome of these trials showed mixed response, some of the studies reported positive effects after RSV supplementation, while others found no significant changes in RSV treated subjects. In patients with diabetic cardiomyopathy, RSV supplementation (250 mg/daily) for 3 months demonstrated improvement in systolic volume, mean arterial blood pressure and blood glucose levels [32]. In another trial, in patients at high risk for cardiovascular disease, daily intake of 8 mg resveratrol for 6 months caused 20% decrease in oxidized LDL cholesterol, but only a modest decrease in LDL cholesterol [33]. Another clinical study tested the efficacy of RSV in MI patients, it was reported that treatment with 10 mg/day of RSV had significantly improved diastolic function with a modest increase in systolic function [34]. On the other hand, in patients with metabolic syndrome, RSV supplementation did not have any effect on systolic and diastolic blood pressure [35,36]. Furthermore, in a recent randomized trial in type II diabetes patients, ingestion of 500mg/day of RSV for 5 weeks failed to show any beneficial effects [37]. Majority of clinical trials were centered on the therapeutic effects of RSV supplementation, whereas studies based on preventive approaches are still lacking. Therefore, in current study, we compared potential preventive and therapeutic role of RSV in an experimental model of Dox induced cardiotoxicity. We found that prophylactic supplementation of RSV is more effective than its therapeutic use in mitigating Dox induced cardiotoxicity in the myocardium.

Doxorubicin is a highly potent anticancer drug, however cardiotoxicity due to Dox limits its use for cancer patients [1,2,38]. Doxorubicin induced cardiac complications have been characterized by thinning and dilatation of the ventricular wall and a reduced ejection fraction. In the present study, we observed a significant reduction in %EF and %FS values in Dox treated animals. Which was associated with an increase in myocardial damage as there was an elevation in CK-MB and LDH levels following Dox administration. In several animal models a similar response has been reported [4,21,22]. Dox induced cardiotoxicity and oxidative damage is reported to be manifested by a significant increase in serum CK-MB levels [21,39]. Doxorubicin induced deterioration of cardiac function was also detected in cancer patients on anti-cancer therapy [40,41]. In our study, co-treatment of RSV with Dox or RSV treatment after 2 weeks of Dox administration were both effective in improving cardiac function. Also there was a significant decrease in myocardial damage after RSV treatment. RSV has been reported to increase myocardial anti-oxidant reserve and free radical scavenging capacity [23,30]. Multiple mechanisms have been reported to be involved in RSV mediated antioxidant reserve. RSV can inhibit nicotinamide adenine dinucleotide phosphate (NADPH) and prevent lipid peroxidation [42,43].

Myocardial apoptosis has been suggested to play a significant role in Dox induced cardiomyopathy and deterioration of heart function. It has been reported that Dox induced oxidative stress lead to activation of intrinsic as well as extrinsic pro-apoptotic pathways in myocardial cells [3–6]. In the current study we observed a significant increase in Bax and a decrease in Bcl-xl as well as an upregulation of caspase 3 expression in Dox group. The Bax protein plays a critical role in intracellular apoptosis. On the other hand, Bcl-xl suppresses apoptosis. Therefore, the ratio of Bax/Bcl-xl determines the susceptibility of a cell to undergo apoptosis. Caspase 3 is also reported to play a central role in the execution of cellular apoptosis [7,8]. Doxorubicin induced oxidative damage lead to alteration in the balance between pro-apoptotic and anti-apoptotic proteins that disturbs mitochondrial membrane permeability and leakage of cytochrome c from mitochondria to the cytosol. In the cytosol cytochrome c binds to apoptotic-protease-activating factor-1 (Apaf-1) that further activates caspase 3 [44,45]. We found that co-treatment with RSV and Dox, as well as RSV treatment after Dox administration decreased the caspase 3 levels in myocardial sections. Interestingly, our studies revealed that prophylactic supplementation of RSV along with Dox was more effective than therapeutic use of RSV in mitigating Dox induced myocardial apoptosis.

It has been previously reported that NFAT family of transcription factors have been involved in Dox induced apoptosis [8]. shRNA mediated inhibition of NFAT3 in glioma cells prevented caspase 3 activation and apoptosis [46]. Doxorubicin treatment activates Ca/calcineurine –NFAT pathway leading to upregulation of FAS/FASL dependent apoptosis [46]. Under basal conditions NFAT3 is present in the cytoplasm in its phosphorylated state. Upon de-phosphorylation at multiple serine residues NFAT3 trans-locates to the nucleus [12,13]. Dox treatment has been reported to mediate translocation of NFAT3 to the nucleus [46,47]. Another NFAT family member NFAT5 is identified as a transcription factor involved in cellular responses to hypertonic stress [48]. NFAT5 regulates expression of several target genes responsible for metabolism of organic osmolytes. Unlike other members of NFAT family, NFAT5 is Ca/calceneurin independent and is regulated by Dox in a different manner [49]. NFAT5 has been reported to play a role in cardiomyocyte survival [49,50]. Previously, Dox induced apoptosis have been suggested to result from proteasome-mediated degradation of NFAT5 [44–51]. In the current study, we found a significant increase in NFAT3 and a decrease in NFAT5 levels in myocardial tissue from Dox treated animals. Resveratrol administration along with Dox or after 2 weeks of Dox treatment decreased NFAT3 and increased NFAT5 expression.

Our study also demonstrate that Dox administration increases myocardial fibrosis as manifested by increased deposition of collagen in the myocardial tissue. Previously it has been reported that Dox induced increase in cardiac fibrosis is associated with upregulation of TGF-ß1 levels [21]. TGF-ß1 is responsible for fibroblasts to myofibroblasts conversion and increase in cardiac fibrosis. Co-treatment with RSV and Dox or RSV treatment after Dox administration decreased Dox induced cardiac fibrosis. In the current study, increase in fibrosis due to Dox administration might be a reparative response by which myocardium compensates for the loss of cells due to Dox induced apoptosis. We did not measure TGF-ß1 levels, however, resveratrol has been previously reported to prevent Dox induced increase in TGF- ß1 and conversion of fibroblasts to myofibroblasts [21,28]. We also found that prophylactic use of resveratrol was more effective than its therapeutic use in mitigating Dox induced fibrosis. Therefore, resveratrol may be used prophylactically as a possible adjuvant therapy to minimize cardio-toxic side effects of Doxorubicin in cancer patients. In conclusion, the outcome of this study will help in interpreting the results of ongoing RSV based clinical trials and carefully design future trials.
